# Mechanical Performances of Isolated Cuticles Along Tomato Fruit Growth and Ripening

**DOI:** 10.3389/fpls.2021.787839

**Published:** 2021-12-17

**Authors:** José J. Benítez, Susana Guzmán-Puyol, Francisco Vilaplana, José A. Heredia-Guerrero, Eva Domínguez, Antonio Heredia

**Affiliations:** ^1^Instituto de Ciencia de Materiales de Sevilla, Centro Mixto Consejo Superior de Investigaciones Científicas-Universidad de Sevilla, Seville, Spain; ^2^Departamento de Mejora Genética y Biotecnología, Instituto de Hortofruticultura Subtropical y Mediterránea “La Mayora”, Universidad de Málaga-Consejo Superior de Investigaciones Científicas, Estación Experimental La Mayora, Málaga, Spain; ^3^Division of Glycoscience, Department of Chemistry, School of Engineering Sciences in Chemistry, Biotechnology and Health, KTH Royal Institute of Technology, Stockholm, Sweden; ^4^Departamento de Biología Molecular y Bioquímica, Instituto de Hortofruticultura Subtropical y Mediterránea “La Mayora”, Universidad de Málaga-Consejo Superior de Investigaciones Científicas, Universidad de Málaga, Málaga, Spain

**Keywords:** tomato fruit cuticles, mechanical characterization, dynamic mechanical analysis, fruit growth and ripening, phenolic compounds

## Abstract

The cuticle is the most external layer that protects fruits from the environment and constitutes the first shield against physical impacts. The preservation of its mechanical integrity is essential to avoid the access to epidermal cell walls and to prevent mass loss and damage that affect the commercial quality of fruits. The rheology of the cuticle is also very important to respond to the size modification along fruit growth and to regulate the diffusion of molecules from and toward the atmosphere. The mechanical performance of cuticles is regulated by the amount and assembly of its components (mainly cutin, polysaccharides, and waxes). In tomato fruit cuticles, phenolics, a minor cuticle component, have been found to have a strong influence on their mechanical behavior. To fully characterize the biomechanics of tomato fruit cuticle, transient creep, uniaxial tests, and multi strain dynamic mechanical analysis (DMA) measurements have been carried out. Two well-differentiated stages have been identified. At early stages of growth, characterized by a low phenolic content, the cuticle displays a soft elastic behavior. Upon increased phenolic accumulation during ripening, a progressive stiffening is observed. The increment of viscoelasticity in ripe fruit cuticles has also been associated with the presence of these compounds. The transition from the soft elastic to the more rigid viscoelastic regime can be explained by the cooperative association of phenolics with both the cutin and the polysaccharide fractions.

## Introduction

The aerial parts of higher plants are covered by the cuticle, a hydrophobic extracellular layer that protects fruits, leaves, seeds, petals, and green stems from the environment. The main functions of the cuticle are to prevent water loss, to regulate the gas exchange, and to act as an effective shield against other external physical agents, such as heat, light, and pressure exerted by insects and pathogens (Domínguez et al., 2011; Heredia-Guerrero et al., 2018).

Fruit firmness and the absence of cracks are features that condition the marketability of many commercial fruits. For this reason, understanding the factors that affect the mechanical endurance of the fruit skin is essential to adopt profitable production and postharvest strategies (Lara et al., 2014). The mechanical integrity of fruits is attributed to the skin (cuticle, epidermis, and hypodermal cell layers; Bargel and Neinhuis, 2005; Khanal and Knoche, 2014). However, most of the biomechanical characterization has been carried out on isolated cuticles, as they mirror the skin performances and constitute a relevant structural component for the integrity of the fruit (Thompson, 2001; Matas et al., 2004a,b; Bargel and Neinhuis, 2005). Currently, there are a number of studies addressing the mechanical characterization of isolated cuticles of fruits and leaves and several comprehensive reviews have been published (Domínguez et al., 2011; Khanal and Knoche, 2017). Thus, parameters, such as stiffness, elastic modulus, rupture stress, and strain as well as the elastic, plastic, and viscoelastic behavior, have been related to the cuticle structure, composition, environmental conditions, and growth stage. For that purpose, procedures, such as uniaxial and biaxial tensile, transient creep and creep-relaxation, and progressive loading and unloading cycle tests, have been performed and reported (Petracek and Bukovac, 1995; Wiedemann and Neinhuis, 1998; Bargel and Neinhuis, 2004, 2005; Matas et al., 2004a,b; Edelmann et al., 2005; López-Casado et al., 2007, 2010; Domínguez et al., 2009; Takahashi et al., 2012; Tsubaki et al., 2012, 2013; Khanal et al., 2013; España et al., 2014; Khanal and Knoche, 2014).

The accuracy and representativeness of these methodologies are conditioned by several factors. Among them: (1) most testing is carried out using isolated specimens, far from the actual *in vivo* stress, hydration, and structural integration conditions; (2) the cuticle is not an isotropic material; in fact, it can be described as a C_16_-C_18_ polyester (cutin) matrix with embedded phenolic compounds and waxes, an important remnant polysaccharide fraction from the cell wall mostly concentrated at the inner side and epicuticular waxes covering the outer region (Domínguez et al., 2011; Yeats and Rose, 2013); and (3) the cuticle thickness is not always uniform since in many species, this continuous layer can infiltrate the anticlinal epidermal cell walls to a variable degree forming wedge-shaped protrusions (pegs). This is common in tomato fruit, where the cuticle can completely encase the epidermis and even extend underneath hypodermal cell layers (Matas et al., 2004a; Bargel and Neinhuis, 2005; Domínguez et al., 2008; Barraj Barraj et al., 2021). As far as key mechanical parameters, such as the Young’s modulus, the rupture stress, and toughness, are calculated considering the cuticle cross-section, the uncertainty in the thickness value compromises an accurate quantification. Despite these difficulties, some general conclusions have been attained. Thus, it has been possible to state the role of water as a plasticizer upon hydration, to elucidate the stiffening effect of waxes and flavonoids acting as fillers, and to relate the elastic and viscoelastic behavior of the cuticle with the polysaccharide and cutin fractions, respectively (Khanal and Knoche, 2017).

The non-elastic (i.e., the viscoelastic and plastic) response of isolated cuticles has been usually obtained from holding or relaxation stages after an applied stress in single or successive creep experiments (Khanal and Knoche, 2017). Often, in these type of experiments, it is difficult to set the limit between the elastic (instantaneous) and non-elastic (time-dependent) regions from the strain-time curves. Additionally, when successive load-unload cycles are used, the final results may be conditioned by an insufficient length of the holding or relaxation period. To avoid these problems, a more refined analytical method, such as dynamic mechanical analysis (DMA), can be employed. In DMA, no time-dependent relaxation or holding is requested and the on-phase strain response to the low amplitude oscillatory stress characterizes the elastic component while the off-phase output corresponds to the viscous element (Menard, 2008). In the literature, there is a limited number of DMA studies on this topic. Some of them were performed with apple cortex tissues (Videcoq et al., 2017) and tomato fruit peels (Wang et al., 2014; Vidyarthi et al., 2020), others with isolated cuticles of persimmon fruits and olive and ivy leaves (Tsubaki et al., 2013; Kamtsikakis et al., 2021). However, to our knowledge, no results using isolated tomato cuticles have been published yet. In this work, the contribution of DMA to the mechanical characterization of a series of isolated cuticles of tomato fruit, collected a different growing stages, is addressed and results are rationalized with those obtained from other techniques. In particular, viscoelasticity has been determined as a function of strain and related to the presence of phenolic compounds along growth and ripening.

## Materials and Methods

### Cuticle Isolation, Composition, and Morphological Analysis

The protocols for sample isolation have been described by España et al. (2014). Briefly, cuticles were enzymatically isolated from *Solanum lycopersicum* “Cascada” using a mixture of fungal cellulase and pectinase in sodium citrate buffer and NaN_3_ to inhibit microbial growth (Petracek and Bukovac, 1995). The cuticles were then separated from the epidermis, rinsed in distilled water, and stored under dry conditions until analysis. The fruit developmental stage is defined as days after anthesis (daa).

### Polysaccharide Analysis

The polysaccharide fraction was isolated after cuticle dewaxing and further cutin degradation. Wax removal was carried out after treating isolated cuticles with a hot chloroform: methanol (2: 1, v:v) mixture for several hours. Cutin degradation was performed in KOH 1% in methanol for 7 days at 40°C. The monosaccharide composition was determined after digestion in trifluoroacetic acid. Briefly, 1 mg sample was weighted accurately and treated with 1 ml TFA 2 M at 121°C for 3 h. Then, 100 μl of solution was diluted until a final volume of 1 ml and filtered through a 0.2 μm PVDF membrane. The monosaccharide analysis was performed using high performance anion exchange chromatography with pulsed amperometry detection (HPAEC-PAD) system (Dionex ICS 3000) equipped with a CarboPac PA1 column. The quantification of monosaccharides was done after calibration with neutral sugars and uronic acid standards at concentrations between 0.005 and 0.1 g/l. The measurements were repeated for 3 replicates. Monosaccharides detected were as: glucose (Glu), xylose (Xyl), manose (Man), glucuronic acid (GlcA), arabinose (Ara), galactose (Gal), and galacturonic acid (GalA). Cellulose content was calculated from the amount of (Glu), while hemicellulose and pectin were the sum of (Xyl + Man+GlcA) and (Ara + Gal+GalA), respectively.

### Infrared Spectroscopy

Attenuated Total Reflected (ATR-FTIR) IR spectra were obtained from both sides of the cuticles using a single reflection ATR accessory (MIRacle ATR, PIKE Technologies, Madison, WI, USA) with a diamond crystal at 45° incidence and coupled to a FTIR spectrometer (FT/IR-6200, Jasco, Tokyo, Japan). All spectra were acquired with a liquid nitrogen cooled MCT detector in the 4,000–600 cm^−1^ range at 4 cm^−1^ resolution and by accumulating 50 scans. Spectrum absorbance was corrected to account for the dependence between the penetration depth and the radiation wavelength in ATR-FTIR measurements. Band areas were calculated using the Jasco SpectraManager software V.2 (Jasco Corporation, Tokyo, Japan).

### Mechanical Characterization

Uniaxial tensile tests of cuticles were conducted using a Criterion 42 (MTS Systems, Eden Prairie, MN, United States) machine equipped with a 10 N load cell and applying a 0.02 N preload. Rectangular uniform pieces (5 mm x 15 mm) were cut and brought to rupture at a constant deformation rate of about 3% min^−1^ at room conditions (~23°C and 45% RH). Samples were inspected with a stereo microscope to check for the absence of cracks. Tomato fruit cuticle changes their color during ripening, reaching an orange-yellow color at red ripe. Given the color variability, within fruits and among them, exhibited in the ripening stages prior to red ripe, samples showing a color representative of the ripening stage were selected. Stress–strain curves were acquired using the specimen cross-section value under no applied load. The cross-section was calculated from the thickness of the continuous cuticle layer on top of the periclinal wall of epidermal cells (t_L_) as observed from optical microscopy. The Young’s modulus was calculated from the maximum slope of the stress–strain curve (typically around 1–2% strain). Experiments were repeated for at least 10 specimens at each ripening stage. Errors in mechanical parameters are expressed as the standard deviation.

Transient creep measurement of the 55 daa Cascada cuticle was performed with a Q800 DMA (TA Instruments, New Castle, DE, United States) and using the tension clamp at room conditions. Sample inspection and dimensions were the same as in uniaxial tensile test. Conditions used, i.e., force increments (∆F) of 0.04 N and holding times (t_H_) of 1200 s were similar to those used in previous studies (López-Casado et al., 2007; España et al., 2014). Here, only the 55 daa sample was measured in transient creep mode to compare with the already published data using a different equipment (España et al., 2014).

Multi strain DMA tests have been done at room conditions with the same analyzer and samples 3 mm wide and with an effective length (distance between grips) of 8 mm. The amplitude of the oscillation was increased from 10 μm in 5 μm increments up to sample rupture. The initial preload force was 0.005 N and the instrument sets an additional static force (Fs) to prevent sample warping. Storage (E') and loss (E'') moduli, as well as their ratio (Tan δ), were recorded as a function of sample strain. Measurements were carried out for 5 specimens at each ripening time. In stages showing evident color variation (35 to 45 daa), samples were sorted in clearer, darker and average and five of each category were analyzed. In addition to the strain evolution of E', E'', and Tan δ, rupture stress and strain were also determined from multi strain DMA tests.

### Statistical Analysis

Regression analysis and the calculation of mean and standard deviation values were performed using OriginPro, Version 2019 software (OriginLab Corporation, Northampton, MA, United States).

### Mechanical Characterization Methods Overview

This section provides a brief description of the methods used for the mechanical characterization of isolated cuticles.

**Figure 1:**
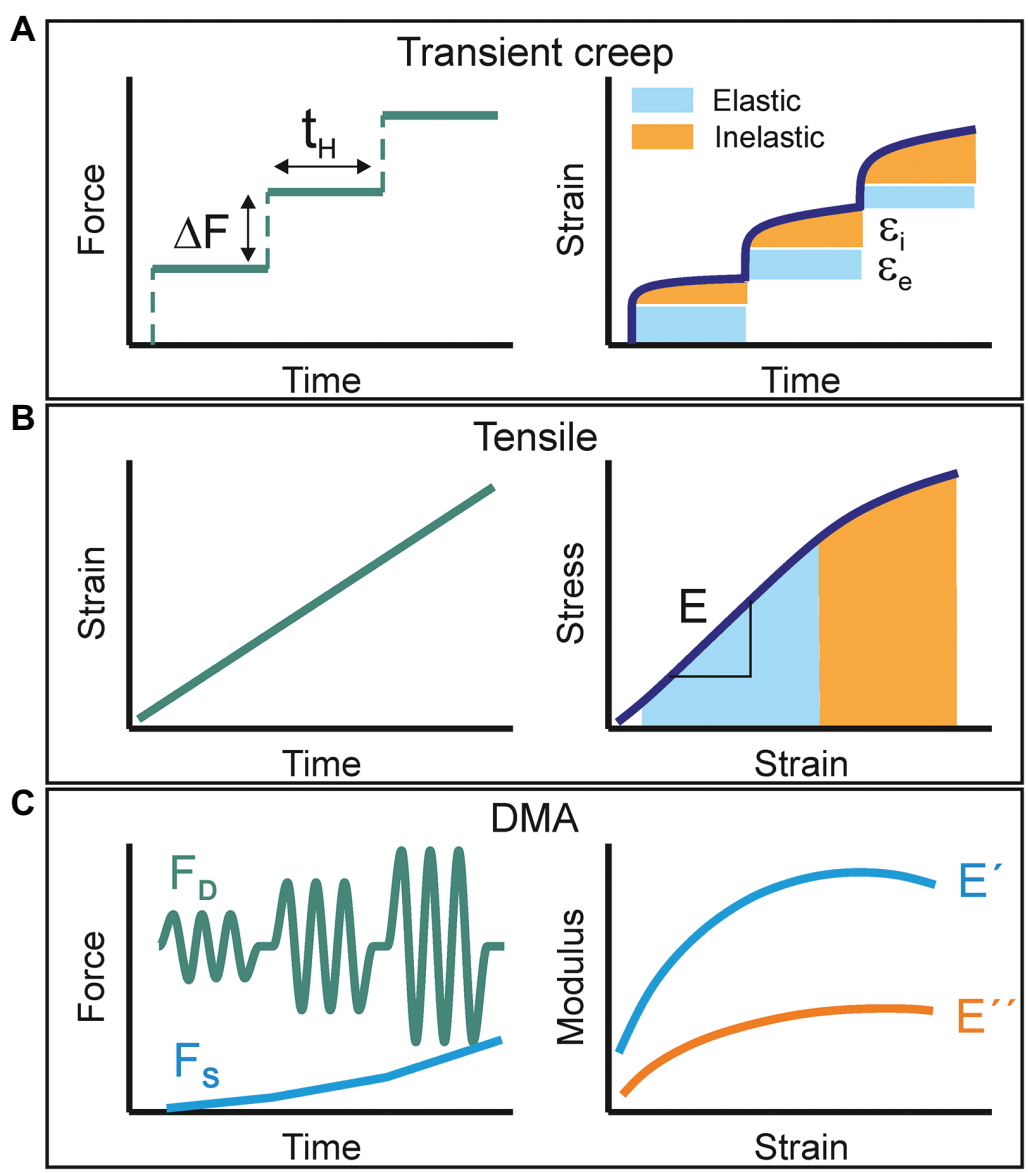
Schematic representation of the mechanical characterization methods used in this study: **(A)** transient creep, **(B)** uniaxial tensile, and **(C)** multi strain dynamic mechanical analysis. The applied perturbation signal is shown on the left side while the collected response is plotted on the right.

Transient creep tests consist in the accumulative application of a force gradient (∆F) for a relatively long holding time (t_H_; Figure 1A). The strain response is then decomposed into an instantaneous (elastic) and a time-dependent (inelastic: viscoelastic + plastic) contributions. At low total forces, the elastic behavior predominates while viscoelastic and plastic deformations progressively show up at higher loads. In addition to the characterization of the elastic and viscoelastic regions of samples, the number of stretch and hold steps can be increased up to sample failure to collect the rupture stress and strain values. However, the main disadvantage of this method is the low productivity. With holding times in the order of several minutes, the number of cycles per time unit is very low. Furthermore, if the value of ∆F is reduced to gain resolution in the corresponding stress–strain curve, the duration of a single experiment may take several hours. When dealing with samples with high variability, such as plant cuticles and to ensure data representativeness, the collection of several replicates significantly slows down obtaining accurate results.

Uniaxial tensile testing is likely the most used procedure to study the mechanical response of films to an applied load. Most of the commercial available instrumentation uses a servomotor to strain the sample at a constant speed and a force transductor to measure the stress exerted to keep such constant deformation rate (Figure 1B). The stress–strain curve of a viscoelastic material commonly displays an initial linear elastic behavior followed by a non-linear segment before failure. The Young’s modulus is calculated as the slope of the linear part, typically around 1% deformation. The area under the curve is the energy per volume needed to break the sample and is an indicator for toughness. Usual strain rates are in the order of several % min^−1^, which leads to experiment duration of few minutes and hundreds of acquired points per stress–strain curve. Test replication is then feasible in a reasonable working time and the productivity and reproducibility of the mechanical characterization improve. However, since there is not a proper static force-time segment, as in creep experiments, it is not possible to accurately define the elastic and inelastic responses.

Dynamic Mechanical Analysis (DMA) is a methodology that combines both the productivity and the capability to characterize the viscoelastic behavior. In a typical DMA experiment, the sample is subjected to a low amplitude sinusoidal stress and the strain response vs. time is monitored. In a pure elastic material, the energy provided by the applied force is stored and released instantly as the stimulus ceases. Consequently, the strain response is synchronized with the stress and their amplitude ratio (stress–strain) is the so-called storage (E') modulus. In a pure viscous material, the energy is fully absorbed (loss) to induce mass flow and there is no instant recovery. A real viscoelastic material can be described by a combination of both models. The total resulting strain response is then delayed (δ) with respect to the oscillating stress and it can be decomposed into an in-phase and an out-of-phase components. The first one provides the pure elastic (storage) modulus (E') and the second one the so-called loss modulus (E”). Their ratio E”/E’ is defined as Tan δ (or damping factor) and constitutes a direct measurement of viscoelasticity. In a DMA experiment, the amplitude of the stress perturbation can be progressively increased (the so-called multi strain mode; Figure 1C). This is achieved by applying a growing dynamic force (F_D_) while a static (F_S_) backup component is added to prevent sample warping and to ensure a periodic response to the oscillation stress. The net applied force can be raised until sample failure and fracture parameters can also be obtained as in transient creep and tensile tests. In this mode, DMA is also capable of evaluating the viscoelasticity (Tan δ) as a function of strain.

## Results

### Physical Parameters and Fraction Composition of Isolated Cascada Tomato Fruit Cuticles

The weight per area (w), density (d), and thickness (t_L_) of the isolated Cascada cuticles as a function of fruit development time (days after anthesis or daa) are compiled in Supplementary Table S1. As observed, the weight per area slightly increases up to 25 daa, stabilizes between 25 and 45 daa, and decreases above 45 daa. The density drops almost to half from 15 to 30 daa and later increases around 30–40% up to 55 daa. The thickness displays a more defined evolution starting with a sharp increment from 15 to 30 daa followed by a progressive reduction above such development time. In general, 30 daa sets a limit for an initial trend involving an increment of weight per area and thickness and a reduction of density. Above 30 daa, these trends reverse but with a less pronounced tendency. These morphological parameters allow the calculation of the ratio between the volume associated to pegs (V_peg_) and that of the uniform and non-invaginated layer (V_L_) of the isolated cuticles according to equation (1). Data in Table 1 show that invagination (V_peg_/V_L_) increases up to a maximum at 25 daa followed by a reduction until 35 daa and a stabilization in the 35–55 daa period.


$$V_{\mathrm{peg}}/V_{L}=\left[w/\left(d\times t_{L}\right)\right]-1$$( 1 ).

**Table 1:** Invagination degree (V_peg_/V_L_) values for tomato Cascada cuticles as a function of fruit development time (daa).

daa (day)	V_peg_/V_L_
15	0.784
20	0.979
25	1.252
30	1.076
35	0.814
40	0.687
45	0.700
50	0.856
55	0.759

The chemical composition of Cascada cuticles along fruit development is shown in Supplementary Table S2. In general, there is not a well-defined evolution of the main fractions (cutin and polysaccharides) and percentages are quite constant. At most, a subtle reduction of the polysaccharide/cutin ratio can be observed at 50 daa and above, which suggests a mild degradation of cell walls at the red-ripe stage. By far, the most noticeable modification during ripening is the accumulation of phenolic compounds. The concentration rapidly increases above 30 daa and it peaks at 55 daa with an order of magnitude increment.

The polysaccharide fraction analysis at selected fruit development times is indicated in Table 2. Samples are quite homogenous in cellulose content though an overall small reduction in hemicellulose and a slight enrichment of pectin can be observed.

**Table 2:** Polysaccharide fraction analysis of cuticles isolated from Cascada tomato fruits harvested at 15, 30, 45, and 55 daa.

daa (day)	Cellulose (%)	Hemicellulose (H) (%)	Pectin (P) (%)	H/cellulose	P/cellulose	(H + P)/cellulose
15	35.5 ± 3.9	40.7 ± 5.7	23.7 ± 3.7	1.15	0.67	1.81
30	36.4 ± 4.4	37.7 ± 4.6	25.8 ± 3.6	1.04	0.71	1.74
45	35.3 ± 4.0	35.9 ± 4.1	28.7 ± 3.0	1.02	0.81	1.83
55	36.1 ± 4.3	36.8 ± 2.9	27.1 ± 2.9	1.02	0.75	1.77

### ATR-FTIR Characterization of Tomato Cascada Cuticles and the Relative Quantification of Phenolic Compounds

**Figure 2:**
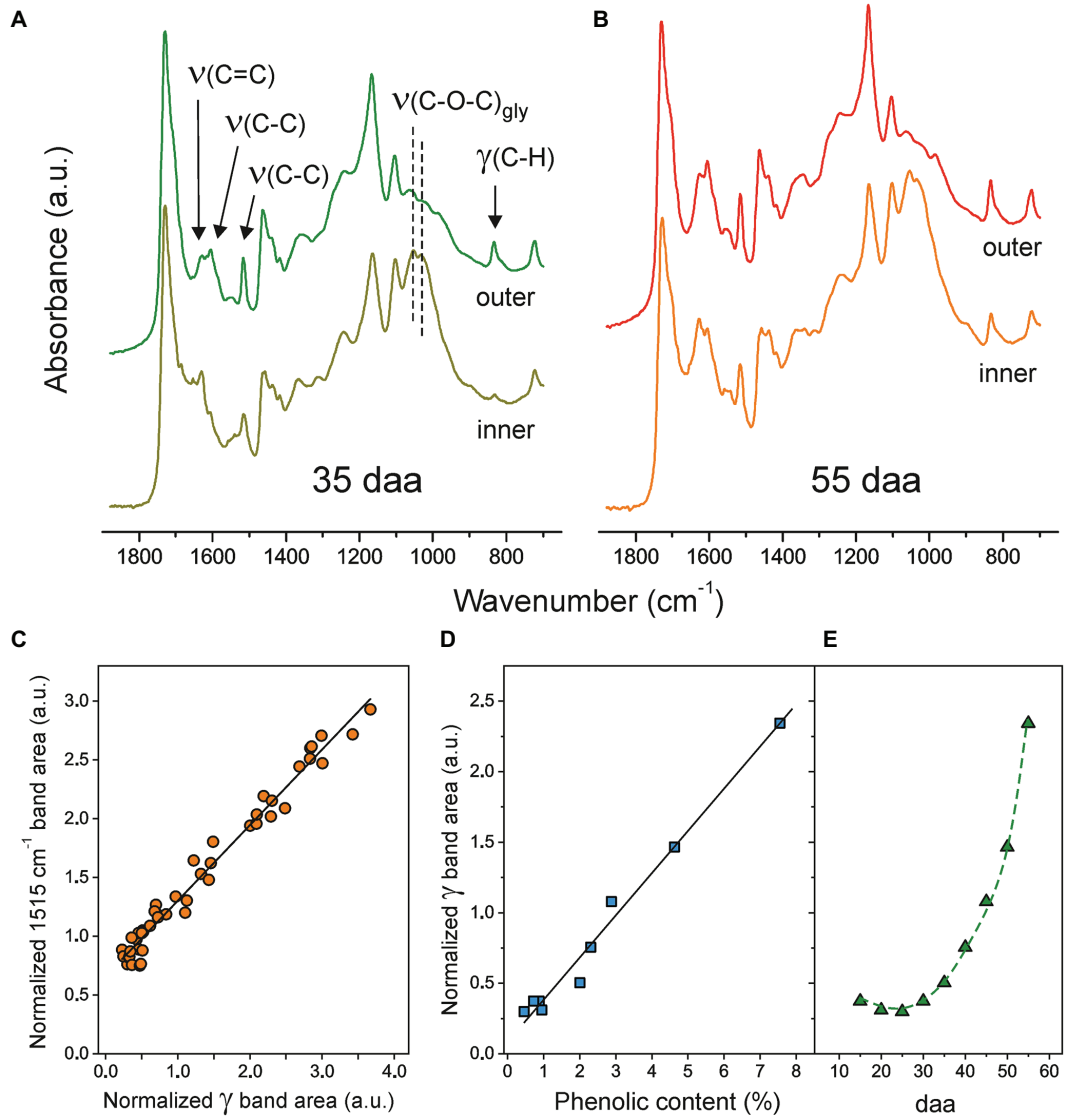
**(A)** and **(B)** Normalized ATR-FTIR spectra (700–1875 cm^−1^) of both sides of Cascada cuticles at 35 and 55 daa, respectively. **(C)** Relationship between normalized areas of bands at 1515 cm^−1^ and (*γ*) at 835 cm^−1^. **(D)** Correlation between the normalized area of the (*γ*) band and the phenolic content obtained from UV–Vis spectroscopy. **(D)** Phenolics evolution in Cascada cuticles along fruit development as monitored by the normalized (*γ*) ATR-FTIR band.

The ATR-FTIR spectra of both, the inner and outer, sides of isolated Cascada cuticles at 35 and 55 daa in the 700–1875 cm^−1^ range are shown in Figures 2A,B. Characteristic ester bands ν(C=O) at 1731 cm^−1^ and ν(C-O-C) at 1168 and 1105 cm^−1^ as well as those of aliphatic chains at 2926 and 2854 cm^−1^ [ν_a_(CH_2_) and ν_s_(CH_2_), respectively, (not shown)] and 1463 and 725 cm^−1^ [scissoring and rocking δ(CH_2_), respectively] are common to both sides and developmental stages. In addition to these peaks (mostly due to cutin), on the inner side, the presence of polysaccharides is revealed by glycosidic ν(C-O-C)_gly_ peaks at 1053 and 1034 cm^−1^ (Heredia-Guerrero et al., 2014). Apart from the accumulation of cutin on the outer side and polysaccharides on the inner surface, the main differences between spectra involve the ν(C=C), ν(C-C), and δ(C-H) modes assigned to aromatic rings in phenolic compounds (Ramírez et al., 1992; España et al., 2014). The evolution of their intensities reveals a noticeable increment of such compounds upon fruit development and their preferential accumulation on the outer regions of the cuticles. The phenolic content in Cascada tomato cuticles throughout development has been reported after cutin depolymerization (España et al., 2014). Here, we propose a new method based on the direct quantification of phenolics ATR-FTIR bands. For this purpose, the ν(C-C) at 1515 cm^−1^ and the so-called gamma (*γ*) band at 835 cm^−1^ have been selected because they are relatively intense, well separated from other absorptions and the base line can be easily defined. However, band intensity in ATR-FTIR is not an absolute measurement and areas should be normalized to allow for the spectra comparison. For normalization, the intense ν(CH_2_) stretching peaks at 2926 and 2854 cm^−1^ have been used as the reference. Figure 2C shows the correlation between the normalized areas of the ν(C-C; 1515 cm^−1^) and *γ* (835 cm^−1^) bands from both sides of the whole series of Cascada cuticles. Despite the good linearity, there is an offset for the 1515 cm^−1^ absorption at zero (*γ*) area. This may indicate that, besides the phenolic compounds, other components may be contributing to the 1515 cm^−1^ peak which invalidate its use for the intended quantitative analysis. Also, when using ATR-FTIR, it has to be considered that its analysis depth is limited. In the experimental conditions used, the effective value is around 4–5 μm, which is below the (t_L_) values for the series (4.4–7.4 μm, Supplementary Table S1). This means that there are regions out of the IR beam penetration range and do not contribute to the spectrum. To overcome this problem and to provide a more precise quantification using ATR-FTIR, the cuticles were analyzed from both sides and the normalized area values averaged. To validate this procedure, results obtained from ATR-FTIR have been compared with those provided by UV–Vis spectroscopy, Figure 2D. As observed, there is a good linearity and no offset at zero coordinate value using the gamma band. Consequently, the normalized (*γ*) ATR-FTIR band area has proven to be an *in-situ*, accurate and fast method to detect the evolution of phenolic content in isolated Cascada cuticles upon fruit development. Such evolution can be observed in Figure 2E: Initially (15–30 daa), the concentration is very low, but it grows very quickly up to a maximum at full maturation (55 daa).

### Comparative Mechanical Characterization of Isolated Tomato Cascada Cuticles

**Figure 3:**
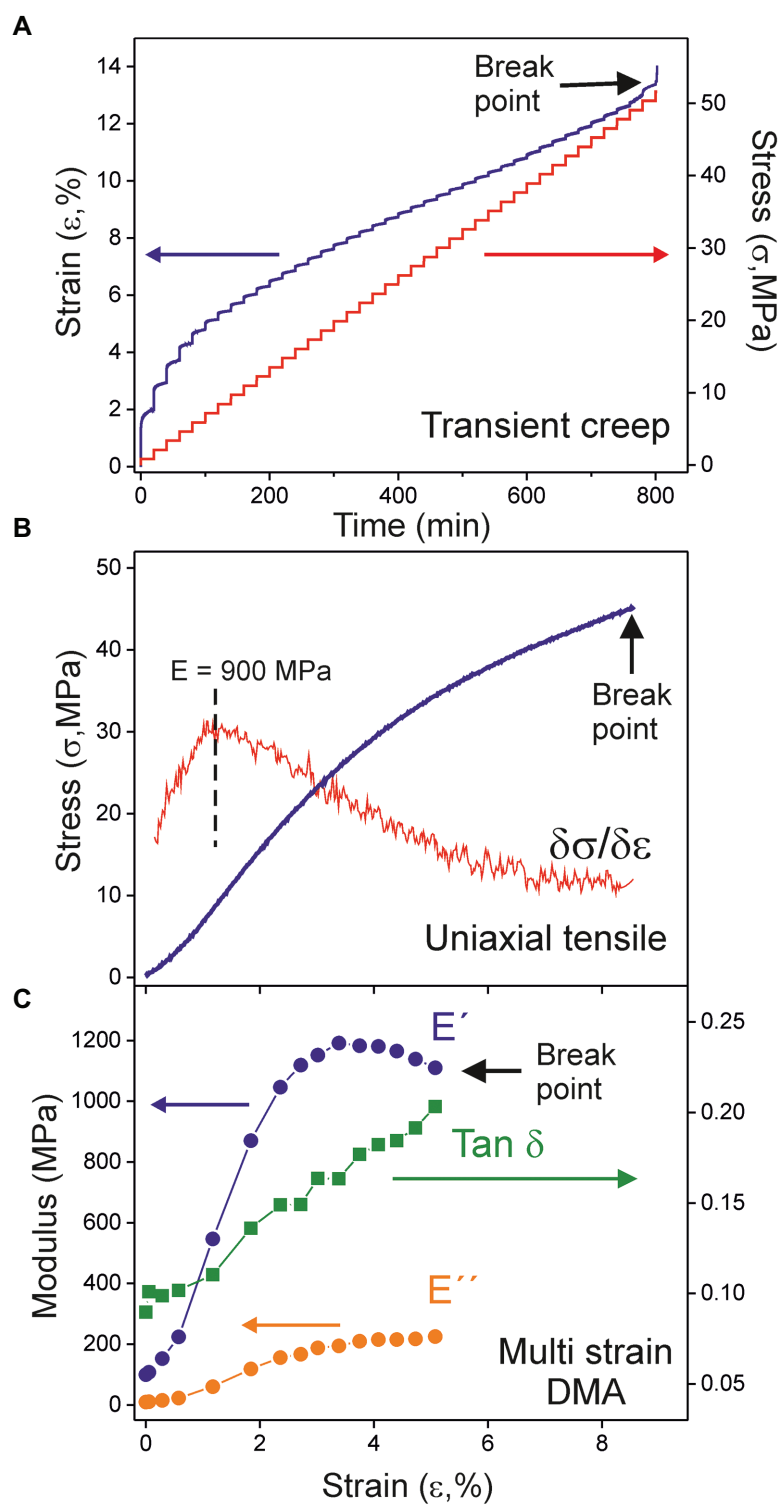
Mechanical analysis of the 55 daa tomato Cascada cuticle by **(A)** transient creep, **(B)** uniaxial tensile, and **(C)** multi strain dynamic mechanical analysis (DMA).

Figure 3 shows the mechanical characterization of the 55 daa cuticle using the three methods above described. The uniaxial tensile test (Figure 3B) has been performed using a universal testing machine while the transient creep (Figure 3A) and the multi strain (Figure 3C) experiments have been collected with the DMA in specific configurations. The transient creep study of the whole Cascada cuticle series has already been reported (España et al., 2014). However, for reproducibility and comparative purposes, the 55 daa has been reanalyzed using the DMA. Results obtained here were a Young’s modulus of 1000 MPa, a breaking stress of 52 MPa and a rupture strain around 13%, which are in good agreement with those reported (884 MPa, 59 MPa, and 14%, respectively) by España et al. (2014).

Figures 3B,C illustrate the general behavior within the Cascada series. The stress–strain curves (Figure 3B) are characteristic for viscoelastic materials, i.e., an initial relatively linear response to the applied force followed by a progressive decay of the slope until sudden rupture. In most cases, stress–strain curves display a subtle “S” shape caused by the lower stress needed to keep the strain rate at the very beginning of the uniaxial test. This effect is likely caused by the fact that cuticle pieces are not macroscopically flat because of the underlying uneven texture of epidermal cells and the inherent curvature of the fruit. Stress is not then uniformly transmitted along the full cross-section of the cuticle and it concentrates in some regions. Consequently, the effective area is smaller than the geometric one and requires less stress to be deformed. As the strain is raised, the area of the effective cross-section increases and stabilizes and the linearity in the mechanical response is regained. Accordingly, the reported Young’s moduli (E) correspond to the maximum slope of the stress–strain curve.

In multi strain DMA tests, both the storage (E') and loss (E'') moduli of cuticles increase with strain until a maximum value is reached, Figure 3C. At higher strain, there is a smooth diminishment before sample break up. This behavior reveals an initial strain-hardening stage followed by a strain-softening one, as previously observed for cuticles of a crack-prone cultivar, such as Sweet 100 (Matas et al., 2004a). In every case, the increment rate of (E'') is higher than (E') as shown by the evolution of their ratio (Tan δ). This result indicates that viscoelasticity increases almost linearly with the strain applied to the cuticles and no well-differentiated limits for the elastic and viscoelastic regions can be stablished.

### Modulus and Rupture Parameters of Tomato Cascada Cuticles

**Figure 4:**
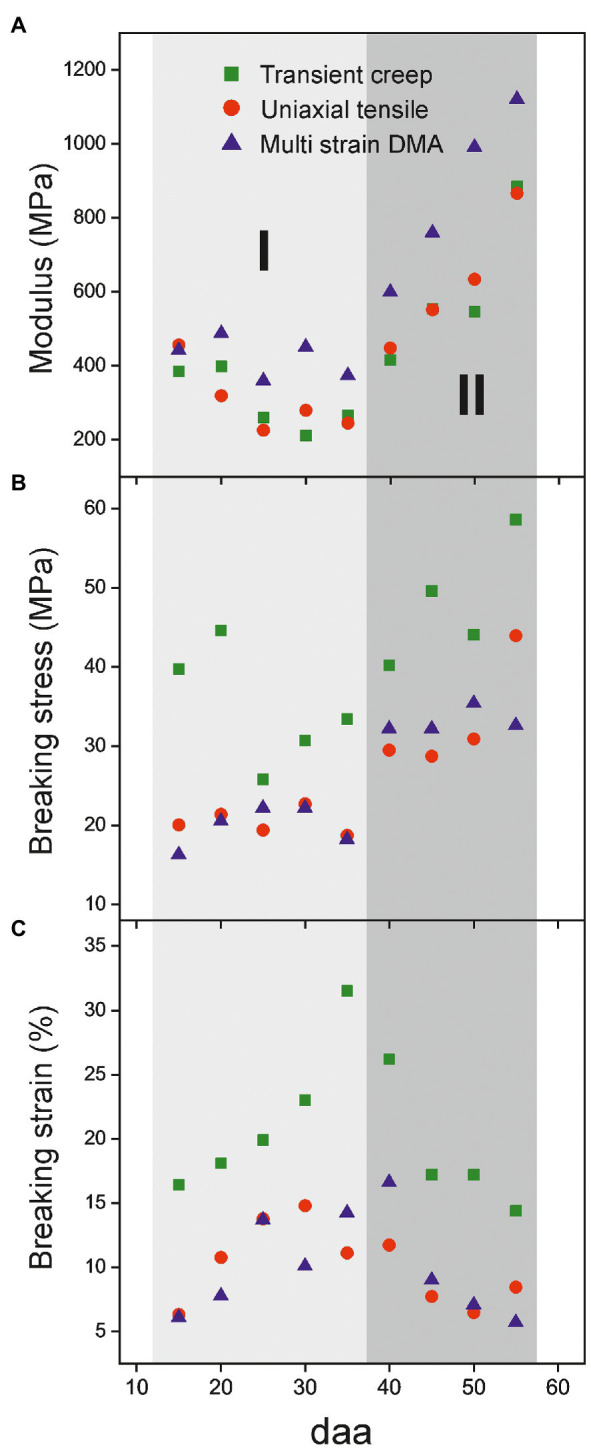
Evolution of mechanical parameters of isolated cuticles along tomato fruit development. **(A)** Modulus, **(B)** breaking stress and **(C)** breaking strain. Two stages (I, 15-35 daa and II, 40-55 daa) can be distinguished.

The modulus, as well as the breaking stress and strain, values are plotted vs. the fruit development time (daa) in Figure 4 (numeric data and errors are provided in Supplementary Table S3). Several peculiarities can be observed from the comparison of the mechanical parameters determined from the three methods used. First, the good concordance between the breaking parameters calculated by tensile and multi strain DMA and the higher values observed in transient creep tests, Figures 4B,C. The most plausible explanation for such difference is based on the long holding times between successive loads in transient creep tests. This procedure adds a viscous extension component to strain and allows the relaxation of local stresses by mass rearrangement and energy dissipation that prevents the formation of a point of fracture and extends the stress limit. Second, the difference between the storage modulus (E') from multi strain DMA and the Young’s modulus from transient creep and uniaxial tensile tests, Figure 4A. Strictly, they are not the same magnitude. The storage modulus (E') is a pure elastic component extracted from a sample subjected to a minimal cyclic stress/relaxation signal with energy being continuously provided and released. Meanwhile, the Young’s modulus is obtained from a unidirectional experiment in which energy is only accumulated by the sample. Besides, it is obtained at larger deformations and it contains a viscous contribution. In this sense, E' can be envisaged as the Young’s value extrapolated to virtually zero strain and, therefore, it is expected to be higher. The same difference between these parameters has been observed for isolated persimmon fruit cuticles (Tsubaki et al., 2013).

Despite the characterization method employed, mechanical parameters of the Cascada series display a reproducible trend along fruit development. In Figure 4, two different stages can be distinguished. Stage I (from 15 to 35 daa) is characterized by a small diminishment of the modulus, an increment of breaking strain and a low, but constant, rupture stress. In stage II (35 to 55 daa), the modification of parameters is more drastic and there is a strong increment of modulus and a noticeable reduction of breaking strain while the breaking stress reaches a maximum plateau within the series.

**Figure 5:**
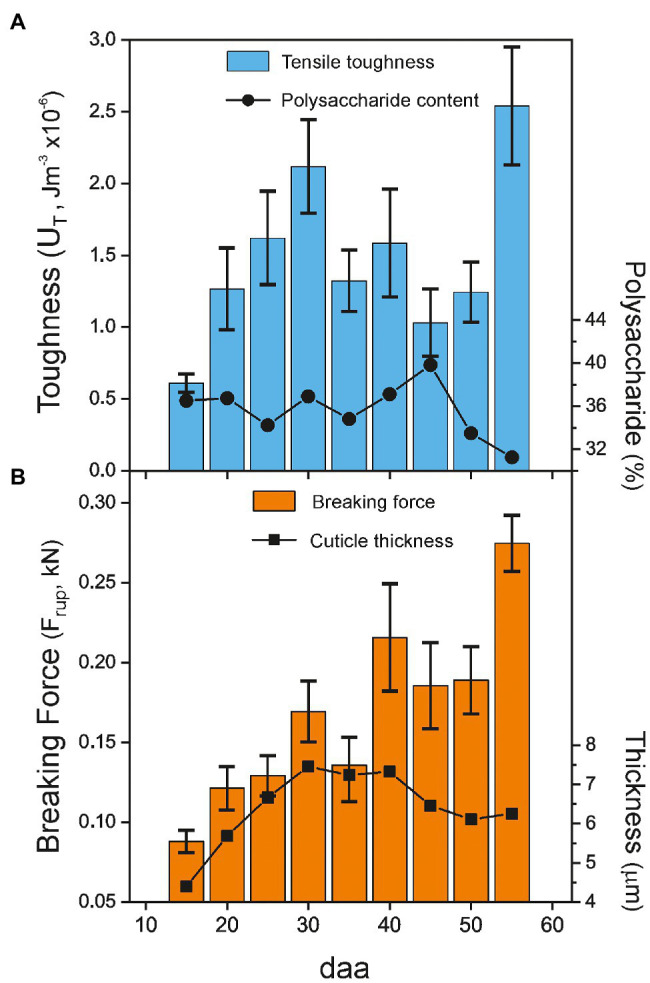
**(A)** Toughness (U_T_) and **(B)** net breaking force (F_rup_) of tomato Cascada cuticles vs. fruit development. For reference, the polysaccharide content (circles) and the cuticle thickness (t_L_; squares) values are included in the plots. Percentage of cuticle polysaccharides and cuticle thickness taken from Domínguez et al. (2008).

Uniaxial tensile testing allows the obtaining of additional interesting mechanical parameters, such as toughness (U_T_) and breaking force (F_rup_). They, respectively, represent the amount of absorbed energy per volume and the force needed for sample breakup. U_T_ is calculated as the area below the stress–strain curve, while F_rup_ is an absolute magnitude directly measured at the point of failure and normalized to 1 m sample width. Figure 5 shows the values for the Cascada series. As observed, there is a sustained growth of toughness from 15 to 30 daa (Figure 5A), mostly caused by the increment in the rupture strain. Above 30 daa, there is not a well-defined behavior due to the opposed evolution of breaking stress and strain values, Figures 4B,C. On the other side, the breaking force increases up to a maximum at 55 daa, Figure 5B. The growth of breaking force in the early stages of development (15–35 daa) seems to be linked to the increment of thickness of the cuticle; however, above 35 daa, the mechanical resistance to rupture improved despite the observed thickness reduction, which suggests the participation of a reinforcing agent surpassing the thickness effect.

### Viscoelastic Behavior of Isolated Tomato Cascada Cuticles

**Figure 6:**
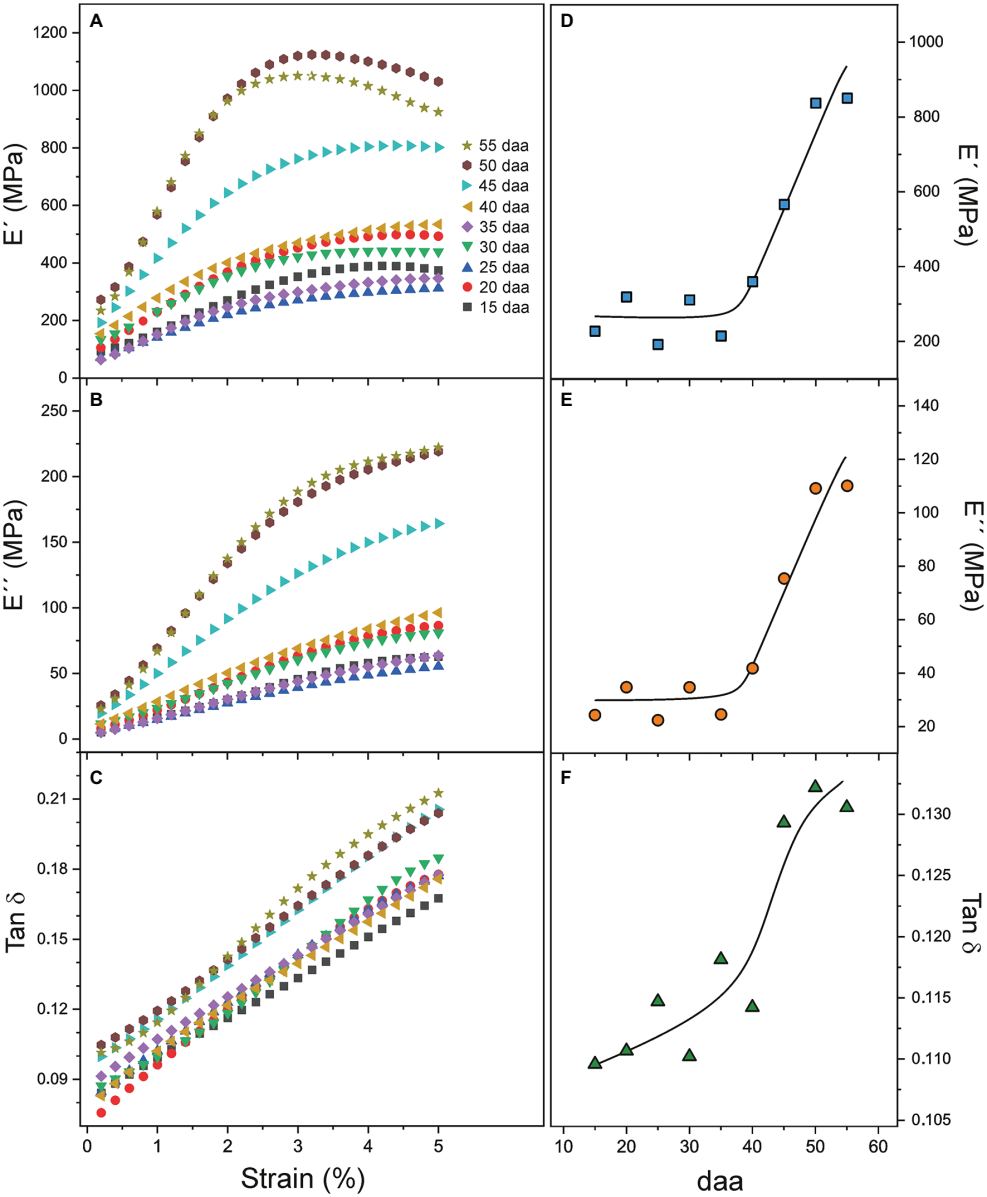
**(A–C)** E', E'', and Tan δ dependence with strain for tomato Cascada cuticles. **(D–F)** E', E'', and Tan δ evolution with ripening time.

Viscoelastic properties of Cascada cuticles have been studied by DMA. Multi strain experiments allow the calculation of the storage (E') and loss (E'') moduli as well as their ratio (Tan δ or damping factor) as a function of strain. Curves for the whole Cascada series are shown in Figures 6A–C. As observed, the shape of the curves depends on the fruit developmental state. The behavior of young, low coloration cuticles is well differentiated from that of ripe ones. Moduli curves within the 15–40 daa period are similar and display a mild increment with strain (strain hardening). At 45 daa and above the evolution of E' and E'' are more pronounced and the growth is clearly associated with ripening time. Tan δ follows a linear trend with strain and the inclination is noticeably incremented at 45 daa and above. Tan δ reveals a linear and progressive increment of viscoelasticity with strain as E'' grows faster than E', but no well-defined transition point between the elastic and viscoelastic behavior of tomato Cascada cuticles is observed.

The evolution of DMA parameters with fruit development time is better appreciated in Figures 6D–F, where E', E'', and Tan δ values at low strain (1.6%) are plotted. Such strain value has been selected because it is below the reported values (2–3%) for the transition between the elastic and viscoelastic regimes in tomato cuticles (Khanal and Knoche, 2017 and references there in) and once the initial non-linear stress–strain stage in cuticles is surpassed (see Figure 3B). Figures 6D–E show the relationship between the moduli and ripening. The initial lower plateau extends from 15 to 35 daa and it is followed by a fast increment up to 55 daa. Tan δ seems to slightly grow from 15 to 40 daa (Figure 6F) and jumps to the highest value in the 45–55 daa period, which indicates a reduction of elasticity and an increment of the viscous component with ripening.

**Figure 7:**
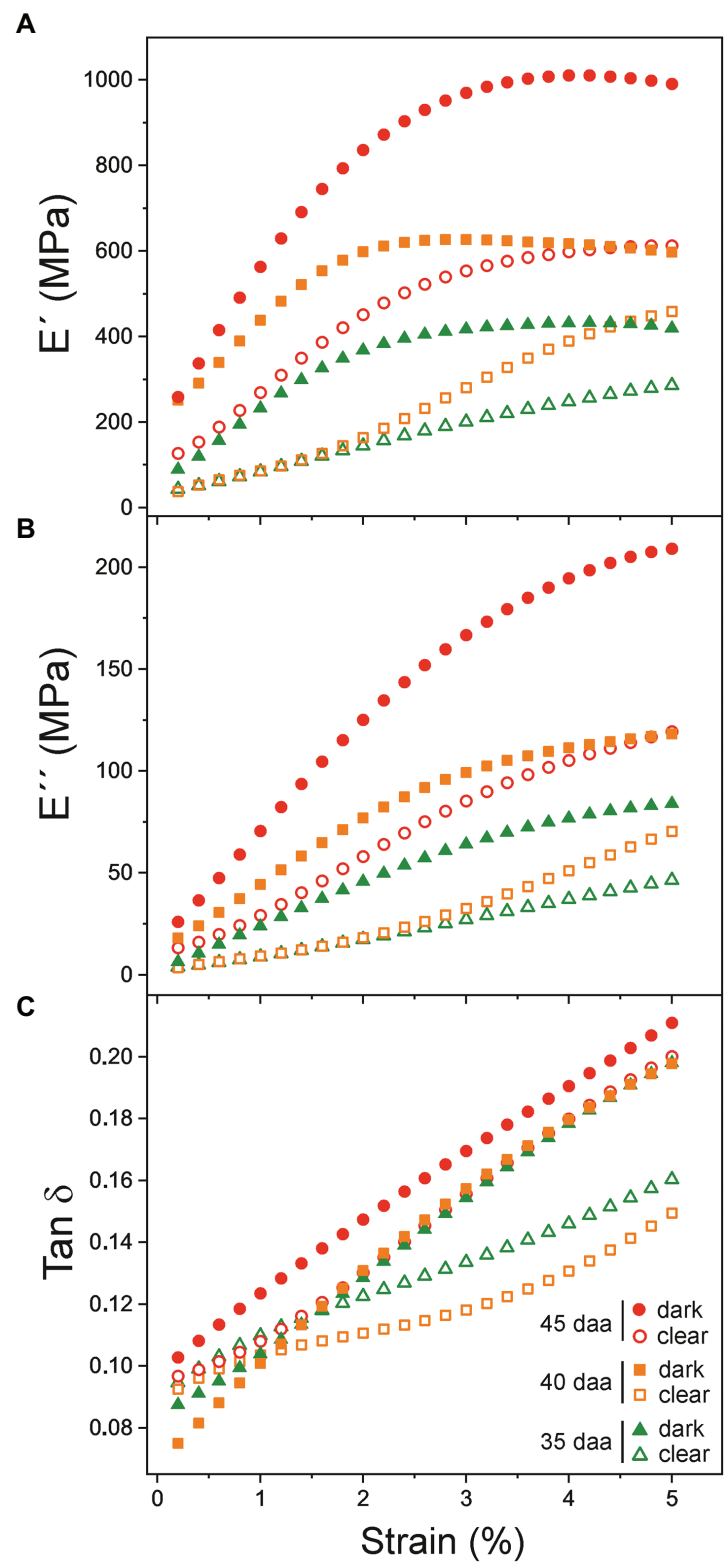
**(A)** Storage (E'), **(B)** loss E'' moduli, and **(C)** Tan δ dependence with strain for cuticles showing differentiated coloration within the same ripening time. The color variability in tomato Cascada cuticles is observed in the 35–45 daa range.

To further investigate the relationship between DMA parameters and the color variability, additional measurements have been performed with cuticles showing a noticeable color variation within a same fruit developmental stage. Visually, samples above (darker) and below (clearer) the average color have been selected for each stage within the 35–45 daa period. E', E'', and Tan δ curves vs. strain are displayed in Figure 7. As noticed, values of clear samples (open symbols) are systematically lower than those of the corresponding darker ones (filled symbols), particularly for younger cuticles (35 and 40 daa). Differences are reduced as ripening progresses to the point that the behavior of clear 45 daa specimens is comparable to the dark set of 35 and 40 daa, while the dark 45 daa resembles the red-ripe stage.

## Discussion

The characterization of isolated tomato Cascada cuticles with a combination of continuous, stepped, and oscillatory mechanical methods has contributed to the elaboration of a more defined model with several stages involving chemical and structural changes along fruit growth and ripening.

### Peg Development Induces Tomato Cuticle Softening at Early Stages of Fruit Growth (Stage I)

The first stage (Stage I) extends from 15 and up to 30–35 daa and it is characterized by a progressive softening, i.e., the reduction of the Young’s modulus and the increment of extensibility before rupture, Figure 4. In this stage, the cuticles are elastic but with slightly growing viscoelasticity, Figure 6F.

Many factors have been reported to affect the mechanical performances of tomato cuticles (Khanal and Knoche, 2017). Among them, the relative amounts of their components. The main fraction of isolated tomato cuticles is cutin and it is generally accepted that cutin increases extensibility and reduces stiffness of cuticles while adding viscoelasticity. The observed evolution of mechanical parameters in tomato cuticles within stage I would be then justified by an increment of the cutin content. However, neither the amount of cuticle per surface area nor the percentage of cutin was reported to change significantly during this period (Domínguez et al., 2008; Supplementary Table S2). Moreover, ATR-FTIR data reveals that there is no reduction of the esterification index in the 15–30 daa range, which rules out the possibility of softening *via* the diminishment of the molecular weight or cross-linking within the cutin polyester matrix due to partial depolymerization.

Quantitatively, polysaccharides are the second component of Cascada cuticles. The presence of the embedded polysaccharides in tomato cuticles has been reported to drastically increase the stiffness of the cutin matrix (López-Casado et al., 2007). Furthermore, the polysaccharide fraction is considered to control the initial elastic stage of the cuticle deformation where the Young’s modulus is calculated. Consequently, it would be reasonable to consider that modifications in the polysaccharide content may induce a change in the elastic response and, thus, the observed cuticle softening in stage I could be tentatively assigned to a reduction of polysaccharides. However, as it was above mentioned for cutin, the percentage of polysaccharides was not reported to change during the 15–30 daa period (Domínguez et al., 2008). Despite the amount of polysaccharides remaining constant, the relative amounts of cellulose, hemicellulose, and pectin displayed small changes, Table 2. The incorporation of pectin and/or hemicellulose to a cellulose matrix has been reported to increase elasticity and extensibility (Chanliaud et al., 2002). However, the observed differences in the relative pectin/cellulose, hemicellulose/cellulose, or pectin+hemicellulose/cellulose ratios (Table 2) are not consistent with the observed 40–45% reduction of the Young’s modulus and the 2-fold increment of the breaking strain in the 15–30 daa stage.

Finally, intracuticular waxes and phenolics have been described as reinforcing fillers in tomato cuticles causing the increment of modulus and rupture strength and the reduction of extensibility (Domínguez et al., 2009; Khanal et al., 2013; España et al., 2014). Yet, as it was the case for the main cuticle components, their reported variation during the 15–30 daa period (Domínguez et al., 2008; España et al., 2014) does not seem to explain the detected tomato cuticle softening.

Data in Supplementary Tables S1, S2 reveal that parameters showing a noticeable modification in stage I (between 15 and 30 daa) are morphological rather than compositional. For instance, cuticle density progressively decreases from 1.665 to 0.908 g cm^−3^ in this period. To explain such reduction, the formation of microscopic and/or nanoscopic cavities along the growth has been suggested (Domínguez et al., 2008). The presence of such cavities would lead to an overestimation of the thickness values (t_L_) used to calculate the Young’s modulus and the breaking stress. Indeed, the modulus reduction in stage I (Figure 3A) would be consistent with such overestimation. Furthermore, the higher elongation at break could also be explained by the formation of a “hollow” structure, as cavities may facilitate the deformation under stress and allow a larger extensibility. However, the breaking stress should also diminish as it is also calculated considering the thickness of the specimen, but the values are quite constant. In other words, there is a direct correlation between the net force needed to break the sample and thickness up to 30 daa (Figure 5B), which is not coherent with the argument of an overestimated thickness caused by void cavities in this growth range. Consequently, other arguments should be provided to explain the tomato Cascada cuticle softening in stage I.

**Figure 8:**
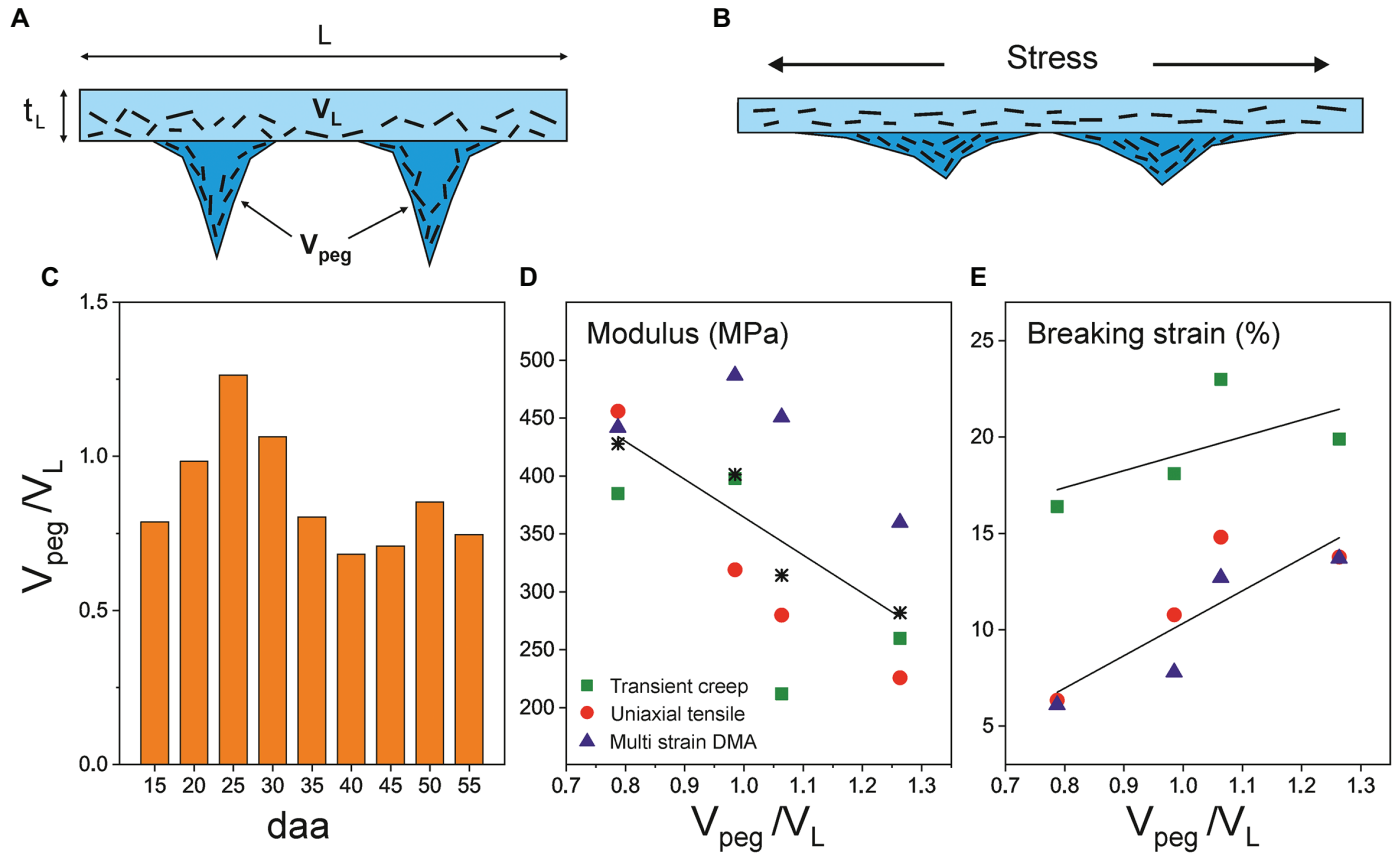
**(A)** Schematic representation of a cross-section of isolated tomato Cascada cuticles and **(B)** under an applied stress. **(C)** Evolution of V_peg_/V_L_ (invagination) along ripening. Correlation between the invagination degree and **(D)** the Young’s modulus and **(E)** the breaking strain in the softening stage (15–30 daa) of tomato Cascada cuticles. Asterisks in **(D)** are the modulus average values.

Figure 8A is a schematic representation of the cross-section of tomato Cascada cuticles based on histological studies (Domínguez et al., 2008). Equation (1) allows the calculation of the ratio between the volumes of the peg and parallel regions (V_peg_/V_L_), which can be regarded as an invagination parameter. Its evolution with fruit growth is represented in Figure 8C. As observed, the invagination generally increases from 15 to 25 daa; then, it slightly decreases up to 35 daa and remains virtually constant up to full ripening. Interestingly, in stage I, and irrespective of the mechanical characterization method used, the modulus correlates negatively with the invagination degree (*p* < 0.01; Figure 8D), while the breaking strain follows the opposite trend (*p* < 0.01; Figure 8E). Matas et al. (2004a) proposed a model in which the rheology of cuticles of tomato cuticles is conditioned by the presence of embedded polysaccharide microfibrils within the cutin matrix. They indicated that the application of a mechanical stress induced the fibril alignment in the direction of the acting force (Figure 8B) which resulted in an increment of the Young’s modulus (strain-hardening). In tomato Cascada cuticles, and according to this model, peg regions would need a higher deformation to rearrange the microfibrils toward a given applied stress resulting in a lower modulus and a higher extensibility. This hypothesis would justify the observed softening in the 15–30 daa period (Stage I) indicating the contribution of peg regions to the overall rheology of the cuticle.

### The Accumulation of Phenolic Compounds Stiffens the Tomato Cuticle (Stage II)

**Figure 9:**
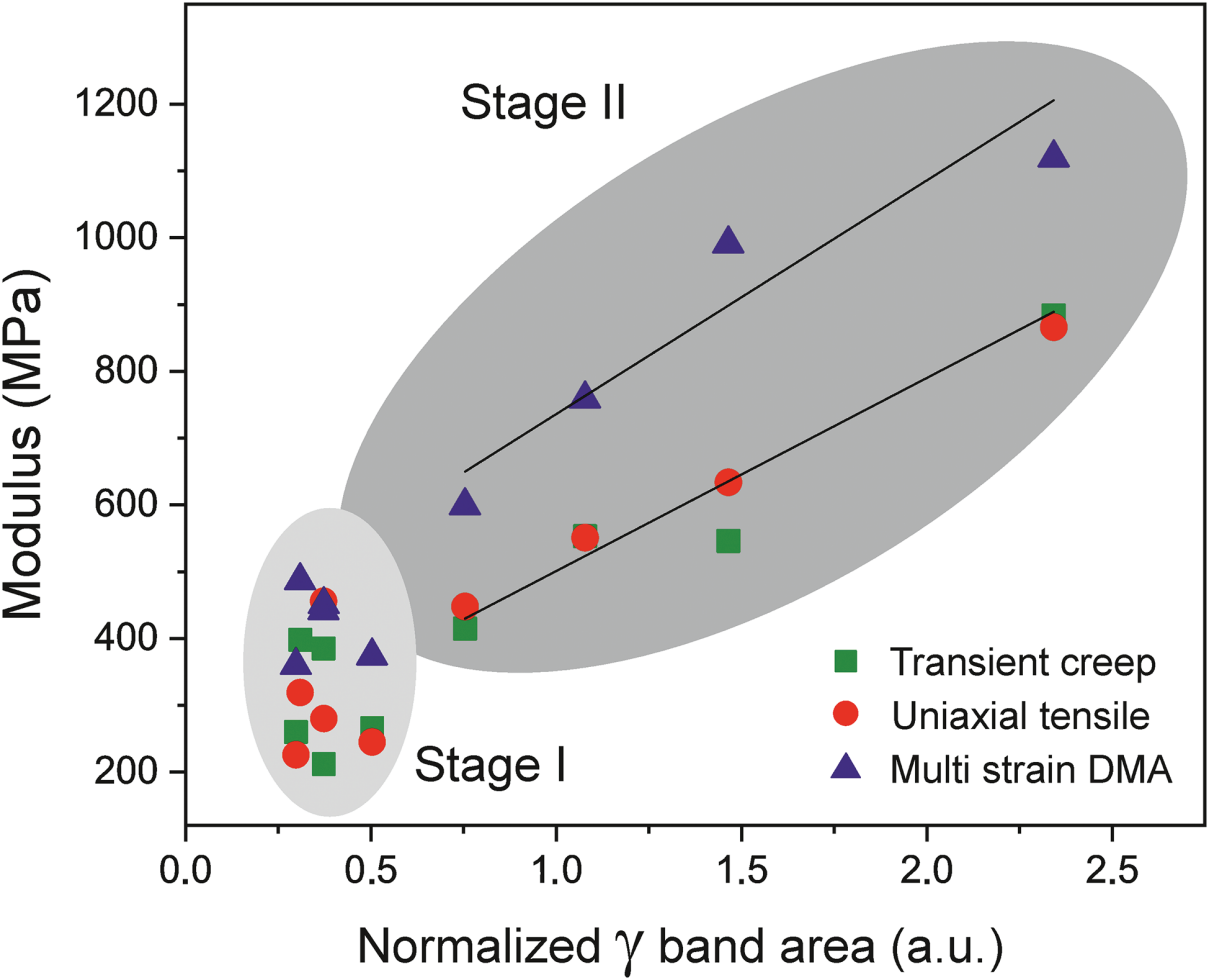
Evolution of Young’s (squares and circles) and storage (triangles) moduli vs. the normalized area of gamma band (phenolics) of cuticles along fruit development. The modulus is correlated with the phenolic content in the 35–55 daa period (Stage II).

Around the onset of ripening, from 35 daa until red ripe (55 daa; Stage II), the mechanical performance of tomato Cascada cuticles changes drastically. They undergo a stiffening process with a noticeable increment of modulus and breaking stress and a reduction of rupture strain, Figure 4. These changes cannot be explained by the modification of morphological traits as invagination and density are relatively constant and the net breaking force increases despite the slight diminishment of thickness, Figure 5B. The cuticle stiffening in the ripening stage (35–55 daa) cannot be associated with the amount and/or type of polysaccharides. Though there is an initial increment of polysaccharide content from 35 to 45 daa (Figure 5A), the amount is significantly reduced at 50 and 55 daa coinciding with the highest cuticle rigidity, Figure 4A. Besides, no meaningful increment of the cellulose and hemicellulose percentages supporting the noticeable increment of modulus is observed from 45 to 55 daa (Table 2). Additionally (see Supplementary Figure S1), no modification of the ATR-FTIR spectra suggesting a modification of the polymerization degree of the polysaccharide fraction has been detected (Castro and Morales-Quintana, 2019). Furthermore, the clear reduction of the esterification index in the 30–55 daa range also discards the possibility of a denser and/or more cross-liked cutin polyester network contributing to the cuticle stiffening. In this scenario, the mechanical behavior of cuticles is univocally conditioned by the accumulation of phenolic species, as previously reported by España et al. (2014). Indeed, a direct correlation (*p* < 0.01) between the modulus and the normalized area of the (*γ*) band can be found for the Cascada series in stage II, Figure 9. Such a direct relationship between the amount of phenolics and the cuticle stiffening can also be found within the same fruit developmental stage when analyzing specimen with variable coloration in the 35–45 daa range (see Supplementary Figure S2). The stage I region falls outside the fit because of the low amount of phenolics and the occurrence of the aforementioned peg-induced softening in such early stage of fruit development.

The stiffening effect of phenolics in tomato cuticles has been associated with their role as molecular fillers in the cutin fraction. Earlier results indicated that phenolics may form clusters trapped in the cutin network (Luque and Heredia, 1994; Laguna et al., 1999) and the affinity of phenolics for the polyester environment is confirmed by ATR-FTIR that shows their accumulation in the outer cutin-enriched regions rather than on the inner polysaccharide-rich fraction of cuticles (Figures 2A,B). The integration of phenolics in the cutin framework is supported by the availability of nanometer size cavities as revealed by computational modeling (Matas and Heredia, 1999) and the induced structural compacting of the cutin network found by XRD (Segado et al., 2020). The rigidity of the cutin matrix has been correlated with the amount of phenolics (López-Casado et al., 2007) and the derived segmental mobility restriction of polyester chains and the reduction of free volume have been evidenced by the increment of the glass transition temperature (*T*_g_; Luque and Heredia, 1997; Matas et al., 2004c). Such interaction of phenolics with cutin and their impact on the mechanical properties of the tomato cuticles is comparable to the effect of the intracuticular triterpenoids on cuticles of persimmon fruits (Tsubaki et al., 2013).

### Phenolics Stiffen Isolated Tomato Cuticles by Interacting With Both the Cutin and the Polysaccharide Fractions

In isolated tomato cuticles, the modulus is the mechanical parameter that experiments the most direct and consistent effect of phenolics. Such parameter is obtained at the elastic regime, which is reported to be conditioned by the polysaccharide fraction and the re-orientation of their microfibrils along the stress direction (Matas et al., 2004a). This consideration would bring the focus on the relevance of polysaccharides on the control of the structural assembly and the mechanical properties of the cuticle (Guzmán et al., 2014; Fernández et al., 2016), a role usually assigned to the polyester cutin. In this sense, it has to be kept in mind that the moduli of tomato cuticles are closer to those of polysaccharides, such as cellulose, hemicellulose, and pectin (in the GPa range) than to cutin and amorphous synthetic cutin-alike polyesters (~10–50 MPa; López-Casado et al., 2007; Benítez et al., 2018). Thus, the massive phenolic incorporation to the cuticle that occurs during ripening could be affecting polysaccharide re-orientation by acting as a compatibilizer on the interface between polysaccharide fibrils and polyester cutin matrix. The potential improvement of polysaccharide-cutin adhesion caused by phenolics would be a shear hindrance to microfibril orientation and a higher stress per deformation unit would be required. Consequently, phenolic compounds may have a new function in cuticles by acting as coupling agents and establishing bridging linkages between polysaccharides and cutin, as proposed for cementing polysaccharides (Fich et al., 2016; Philippe et al., 2020).

**Figure 10:**
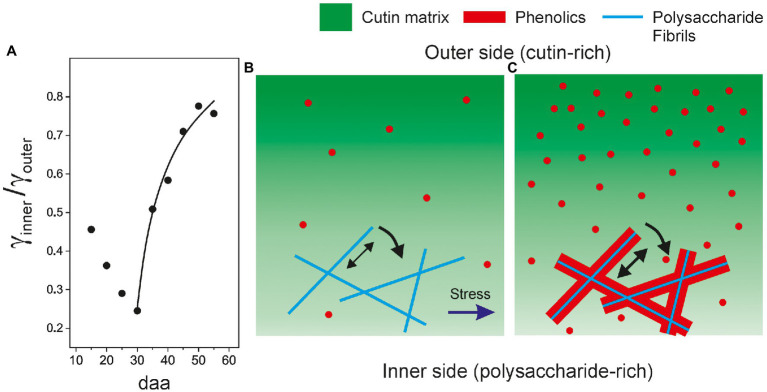
**(A)** Evolution of the ratio of normalized (*γ*) bands as obtained from the ATR-FTIR spectra from the inner (*γ*_inner_) and outer (*γ*_outer_) sides of tomato Cascada cuticles. Structural model showing the fractions (cutin, polysaccharides, and phenolics) distributions in: **(B)** young and **(C)** ripe cuticles.

A closer look to ATR-FTIR data supports the proposed association of phenolics with the polysaccharide fraction. In Figure 10A, the ratio between the normalized (*γ*) band areas corresponding to the inner (polysaccharide-rich) and outer (cutin-rich) sides of Cascada cuticles is plotted versus fruit development time time. As observed, there is a noticeable increment of the *γ*_inner_/*γ*_outer_ ratio matching the phenolic accumulation stage (30 to 55 daa). The thickness and invagination variations in this range are small (Table 1; Supplementary Table S1) and, consequently, data should be considered to be free of the inherent thickness distortion of ATR-FTIR measurements (see Additional Text in Supplementary Material). The interpretation of the *γ*_inner_/*γ*_outer_ increment is that, during ripening in stage II, the rate of accumulation of phenolics in the polysaccharide-rich region is faster than in the cutin-rich one, as expected from the proposed phenolics-polysaccharide association. The initial *γ*_inner_/*γ*_outer_ diminishment observed between 15 and 30 daa is very likely a thickness artifact (see Additional Text in Supplementary Material).

The structural model proposed here is schematized in Figures 10B,C and combines two effects caused by phenolics: (1) their association with polysaccharide fibrils and the increment of adhesion between fibrils and cutin and (2) the stiffening of the cutin matrix resulting from their accommodation as nanofillers. Since tomato fruit cuticle phenolics are mainly phenolic acids, which are present throughout development but significantly increase their accumulation during ripening, and the flavonoid chalconaringenin, that is only incorporated to the cuticle during ripening (España et al., 2014), it would be important to ascertain whether both phenolic compounds can act as nanofillers and compatibilizers.

### Viscoelasticity of Isolated Tomato Cuticles Is Modulated by the Presence of Phenolic Compounds

The evolution of the storage modulus (E') with strain displays an initial increment (strain-hardening; Figure 6A) caused by the re-orientation of fibrils along the stress direction. At low phenolic content, the interfacial adhesion between polysaccharides and cutin is comparatively low and, consequently, the re-orientation requires a low stress. As phenolics accumulate, they associate with polysaccharides and the cutin-polysaccharide interaction gets reinforced. The higher interfacial adhesion between fibrils and cutin, as well as the stiffening of the cutin itself, caused by phenolics entail a higher stress for the re-orientation and the storage modulus (E') increases significantly (Figures 6A,D). At higher strains, the strain-hardening is followed by a strain-softening assigned to the slippage of polysaccharide fibrils within a plastic deformed cutin matrix (Spatz et al., 1999; Köhler and Spatz, 2002). The transition between both regimes sets a (E') maximum which is also affected by the fruit developmental stage. The maximum is higher, more pronounced and displaced toward lower strains upon phenolics irruption because the stress required for the orientation of fibrils raises so quickly that reaches the slippage limit at lower strain values. The cuticle stiffening induced by these compounds is so intense that compensates and surpasses the effect of the polysaccharide reduction in the 45–55 daa range (Figure 5A). Indeed, evaluation of the effect of phenolics on the elastic modulus was shown to be an order of magnitude higher than the one of polysaccharides (Domínguez et al., 2009).

It has been suggested that the predominance of the viscoelastic performance of cuticles is related to the absence of phenolics (Domínguez et al., 2009). This conclusion is based on the analysis of the shape of the stress–strain curve in transient creep experiments. However, DMA analysis is capable to isolate the pure elastic and viscous components from the very beginning of deformation. Thus, Figure 6C shows that the viscoelasticity (Tan δ) increases with the phenolic content even within the elastic region (ε < 2–3%). This behavior can be due to a higher contribution of the viscous component arising from a better compatibilization between polysaccharides and cutin phases favored by phenolics. Nevertheless, an effect of the polysaccharide decrease observed in the 45–55 daa period cannot be dismissed (López-Casado et al., 2007).

The increased accumulation of phenolic compounds in tomato cuticle during ripening allows the transition from a soft elastic regime to a more rigid and viscous state. Such rheological modification is relevant to the understanding of key phenomena, such as mass flow, diffusion, and transpiration processes across this barrier layer. The association of phenolics with polysaccharides and its contribution to the modification of the biomechanical performance of tomato cuticles are issues that deserve further research.

## Data Availability Statement

The raw data supporting the conclusions of this article will be made available by the authors, without undue reservation.

## Author Contributions

JB, ED, and AH conceived and planned the research. JB performed the experiments. SG-P and FV were responsible for the polysaccharide analysis. JH-G supervised the experiments. JB, JH-G, and ED wrote the article with contributions and supervision of all authors. All authors approved the submitted text.

## Funding

This work was supported by grant RTI2018-094277-B/AEI/10.13039/501100011033 from Agencia Estatal de Investigación, Ministerio de Ciencia e Innovación, Spain (co-financed by the European Regional Development Fund, ERDF). JH-G acknowledges the support by the Spanish “Ministerio de Ciencia, Innovación y Universidades” project RYC2018-025079-I/AEI/10.13039/501100011033 (co-financed by the European Social Fund, ESF). SG-P thanks the support of the PIE project 202040E003 funded by the Spanish Research Council (CSIC).

## Conflict of Interest

The authors declare that the research was conducted in the absence of any commercial or financial relationships that could be construed as a potential conflict of interest.

## Publisher’s Note

All claims expressed in this article are solely those of the authors and do not necessarily represent those of their affiliated organizations, or those of the publisher, the editors and the reviewers. Any product that may be evaluated in this article, or claim that may be made by its manufacturer, is not guaranteed or endorsed by the publisher.
