# Supplementary material for: Mechanical Performances of Isolated Cuticles Along Tomato Fruit Growth and Ripening
Source: Front Plant Sci. 2021 Dec 17;12:787839. doi: 10.3389/fpls.2021.787839 (PMC8718444; doi:10.3389/fpls.2021.787839)
Supplement: Supplementary file 1 [file Data_Sheet_1.PDF]

SUPPLEMENTARY INFORMATION

Mechanical Performances of Isolated Tomato Cuticles Along Fruit Ripening

José J. Benítez[1], Susana Guzmán-Puyol[2], Francisco Vilaplana[3], José A. Heredia-Guerrero[2], Eva Domínguez[2] and Antonio Heredia[4]*

[1] *Instituto de Ciencia de Materiales de Sevilla, Centro Mixto Consejo Superior de Investigaciones Científicas-Universidad de Sevilla, Seville, Spain.*

[2] *Instituto de Hortofruticultura Subtropical y Mediterránea "La Mayora", Universidad de Málaga-Consejo Superior de Investigaciones Científicas, Departamento de Mejora Genética y Biotecnología, Estación Experimental La Mayora, Málaga, Spain*

[3] *Division of Glycoscience, Department of Chemistry, School of Engineering Sciences in Chemistry, Biotechnology and Health, KTH Royal Institute of Technology, Stockholm, Sweden*

[4] *Instituto de Hortofruticultura Subtropical y Mediterránea "La Mayora", Universidad de Málaga-Consejo Superior de Investigaciones Científicas, Departamento de Biología Molecular y Bioquímica, Universidad de Málaga, Málaga, Spain*

Supplementary Table S1. Weight per area (w), density (d) and thickness of the non-invaginated region (t_L) for tomato Cascada cuticles as a function of fruit ripening time (daa).

daa (day)	Weight (w) (μg cm^{-2})	Density (d) (g cm^{-3})	Thickness (t_L) (μm)
15	1307	1.665	4.4
20	1346	1.193	5.7
25	1456	0.965	6.7
30	1395	0.908	7.4
35	1446	1.107	7.2
40	1367	1.110	7.3
45	1443	1.306	6.5
50	1253	1.107	6.1
55	1332	1.221	6.2

Data are extracted from Domínguez, E., López-Casado, G., Cuartero, J. and Heredia, A. (2008). Development of fruit cuticle in cherry tomato (*Solanum lycopersicum*). *Funct. Plant Biol.* 35, 403-411. doi: 10.1071/FP08018 and España, L., Heredia-Guerrero, J. A., Segado, P., Benítez, J. J., Heredia, A. and Domínguez, E. (2014). Biomechanical properties of the tomato (*Solanum lycopersicum*) fruit cuticle during development are modulated by changes in the relative amounts of its components. *New Phytol.* 202, 790-802. doi: 10.1111/nph.12727

Supplementary Table S2. Fraction composition (in %, w/w) of isolated Cascada cuticles along fruit ripening. The 100% is set on the basis of the cutin, polysaccharides and total wax contents. Phenolics percentage is referred to naringenin as a standard. The esterification index (E.I.) is defined as the ATR-FTIR $\nu(C{=}O)/\nu_a(CH_2)$band area ratio.

Daa (day)	Wax (total) (%)	Wax (epi) (%)	Wax (intra) (%)	Cutin (%)	Polysaccharides (%)	Phenolics (%)	E.I.
15	2.40	0.39	2.01	61.09	36.51	0.88	1.32
20	3.64	1.21	2.43	59.63	36.73	0.95	1.33
25	3.52	1.10	2.42	62.23	34.25	0.46	1.34
30	2.45	0.76	1.69	60.64	36.91	0.72	1.33
35	2.98	1.13	1.85	62.22	34.80	2.00	1.27
40	3.28	1.13	2.15	59.60	37.12	2.30	1.18
45	3.36	1.08	2.28	56.81	39.83	2.88	1.13
50	3.25	1.15	2.10	63.25	33.50	4.62	1.08
55	2.83	0.57	2.26	65.91	31.26	7.55	1.05

Data are extracted from Domínguez, E., López-Casado, G., Cuartero, J. and Heredia, A. (2008). Development of fruit cuticle in cherry tomato (*Solanum lycopersicum*). *Funct. Plant Biol.* 35, 403-411. doi: 10.1071/FP08018 and España, L., Heredia-Guerrero, J. A., Segado, P., Benítez, J. J., Heredia, A. and Domínguez, E. (2014). Biomechanical properties of the tomato (*Solanum lycopersicum*) fruit cuticle during development are modulated by changes in the relative amounts of its components. *New Phytol.* 202, 790-802. doi: 10.1111/nph.12727

Supplementary Table S3. Mechanical parameters for isolated tomato Cascada cuticles using the indicated characterization techniques. (E)Young´s modulus, (E´) maximum storage modulus, **(**σ) rupture stress and (ε) rupture strain.

daa	Transient creep (*)			Tensile tests			Multi strain DMA		
	E (MPa)	σ (MPa)	ε (%)	E (MPa)	σ (MPa)	ε (%)	E´ (MPa)	σ (MPa)	ε (%)
15	385±55	40±5	16±3	456±25	20±3	6±1	442±52	16±8	6±2
20	398±32	45±3	18±2	319±56	21±5	11±4	487±70	21±9	8±3
25	260±18	26±5	20±5	226±50	19±4	14±5	360±38	22±6	14±4
30	212±32	31±2	23±3	280±51	23±5	15±5	451±34	22±6	13±2
35	266±31	33±2	32±2	245±39	19±6	13±4	374±53	18±7	14±4
40	415±34	40±3	26±1	448±59	29±6	12±5	599±28	32±7	17±5
45	553±12	50±1	17±1	551±74	29±8	8±2	759±72	32±8	9±4
50	546±32	44±3	17±3	634±81	31±9	7±1	991±110	35±7	7±3
55	884±70	59±7	14±3	866±81	44±10	8±3	1120±81	33±4	6±1

(*) Extracted from España, L., Heredia-Guerrero, J. A., Segado, P., Benítez, J. J., Heredia, A. and Domínguez, E. (2014). Biomechanical properties of the tomato (*Solanum lycopersicum*) fruit cuticle during development are modulated by changes in the relative amounts of its components. *New Phytol.* 202, 790-802. doi: 10.1111/nph.12727

Supplementary Figure S1. ATR-FTIR spectra of the inner side (polysaccharide rich) of isolated tomato cuticles along the ripening stage (35-55 daa). Spectra similarity as well as the negligible band shift (<2cm^{-1}) indicate the homogeneity of the polysaccharide fraction along the ripening process.

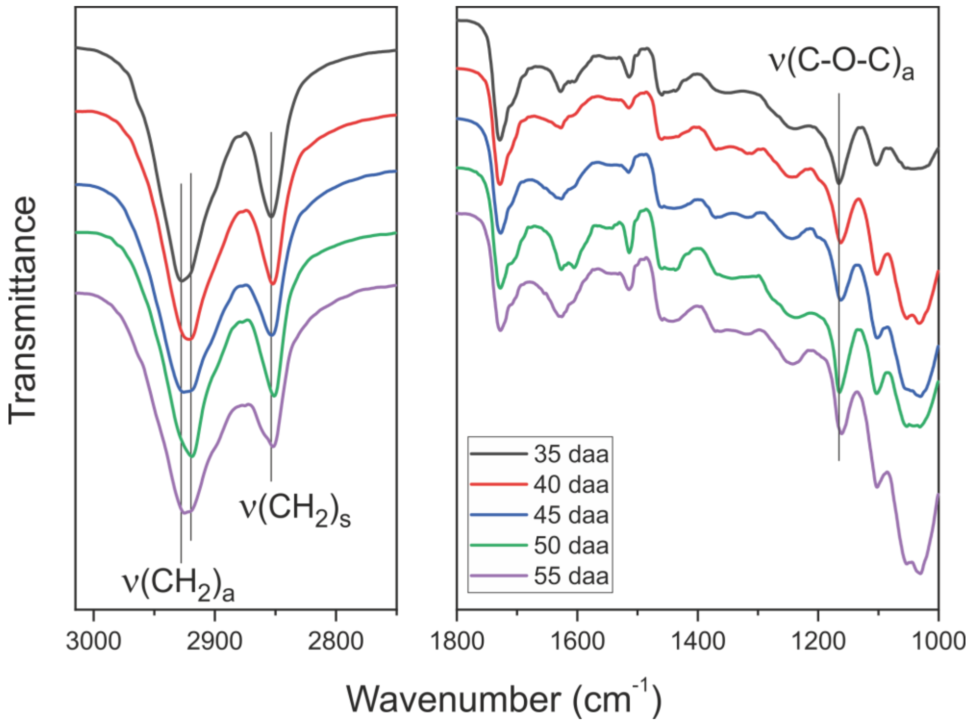

Supplementary Figure S2. Relationship between the Young´s modulus and the amount of phenolics (γ band area) in tomato cuticles within the same developmental stage of the fruit (daa). For this purpose, cuticles have been visually sorted into below, above and average coloration groups and the modulus and the normalized γ band area values obtained for each group have been averaged. Systematically, at every developmental stage, the increment of color intensity is accompanied by the increase of γ band area.

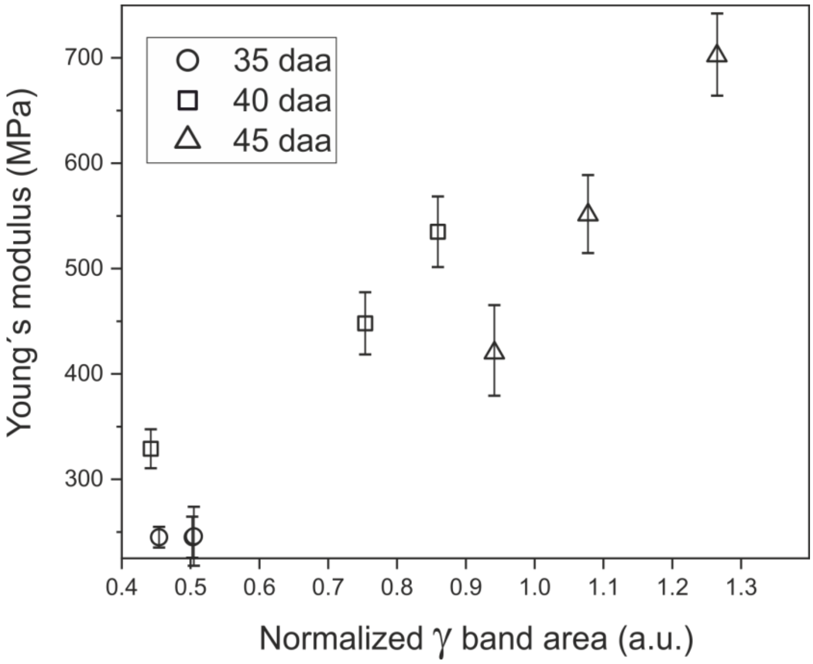

Additional Text

To understand data in **Figure 10A**, a couple of considerations should be taken into account. First, ATR-FTIR cannot provide quantitative data on the amount of phenolic compounds in cuticles using absolute band areas. The only way to compare data from different spectra is to use relative values and, for that reason, the gamma band is referred to the intense methylene stretching signal mostly arising from the polyester cutin. Thus, what the relative (γ) band area provides is the amount of phenolics in relation to the amount of cutin within the sample volume accessed by the IR beam. The phenolics/polysaccharide ratio cannot be easily obtained (polysaccharide peaks overlap with others) and cannot be compared with the phenolics/cutin ratio because of the different sensitivity factor of their corresponding ATR-FTIR bands. Second, ATR-FTIR is a surface sensitive technique that gathers information from a limited region beneath the irradiated surface, consequently, a distortion in the composition profile will be present when comparing cuticles with different thickness. The initial diminishment observed between 15 and 30 daa in **Figure 10A** is an artifact because, in this range, the thickness and invagination grows continuously. In thinner samples the ($\gamma_{inner}/\gamma_{outer}$) ratios are prone to get closer to 1 because the entire cuticle is virtually analyzed independently from the side that is being irradiated. As the cuticle gets thicker ($\gamma_{inner}/\gamma_{outer}$) values are more realistic and the non-isotropic distribution of fractions becomes more evident in ATR-FTIR measurements, as is the case for the 30-55 daa range.
